# Recovery of Flavonoids from Orange Press Liquor by an Integrated Membrane Process

**DOI:** 10.3390/membranes4030509

**Published:** 2014-08-11

**Authors:** Alfredo Cassano, Carmela Conidi, René Ruby-Figueroa

**Affiliations:** Institute on Membrane Technology, ITM-CNR, c/o University of Calabria, via Pietro Bucci, 17/C, I-87036 Rende (CS), Italy; E-Mails: c.conidi@itm.cnr.it (C.C.); r.ruby@itm.cnr.it (R.R.-F.)

**Keywords:** orange press liquor, ultrafiltration (UF), nanofiltration (NF), osmotic distillation (OD), integrated membrane processes, flavonoids

## Abstract

Orange press liquor is a by-product generated by the citrus processing industry containing huge amounts of natural phenolic compounds with recognized antioxidant activity. In this work, an integrated membrane process for the recovery of flavonoids from orange press liquors was investigated on a laboratory scale. The liquor was previously clarified by ultrafiltration (UF) in selected operating conditions by using hollow fiber polysulfone membranes. Then, the clarified liquor with a total soluble solids (TSS) content of 10 g·100 g^−1^ was pre-concentrated by nanofiltration (NF) up to 32 g TSS 100 g^−1^ by using a polyethersulfone spiral-wound membrane. A final concentration step, up to 47 g TSS 100 g^−1^, was performed by using an osmotic distillation (OD) apparatus equipped with polypropylene hollow fiber membranes. Suspended solids were completely removed in the UF step producing a clarified liquor containing most part of the flavonoids of the original press liquor due to the low rejection of the UF membrane towards these compounds. Flavanones and anthocyanins were highly rejected by the NF membrane, producing a permeate stream with a TSS content of 4.5 g·100 g^−1^. An increasing of both the flavanones and anthocyanins concentration was observed in the NF retentate by increasing the volume reduction factor (VRF). The final concentration of flavonoids by OD produced a concentrated solution of interest for nutraceutical and pharmaceutical applications.

## 1. Introduction

World citrus production and consumption have grown strongly since the mid-1980s. Brazil is the world’s leading orange producer (about 28% of the world’s production) with a production of 18 million tons in 2012 [1] followed by the United States and European Union.

Oranges are commonly peeled and eaten fresh or squeezed for juice. More than 80% of citrus fruit production is transformed into juice; residues generated in this step consist basically in wet peels and whole rejected fruits containing, on average, 82% water [2]. These semi-solid wastes can be pressed to obtain a liquid, named press liquor, which is a complex mixture containing soluble sugars (sucrose, glucose and fructose), insoluble carbohydrates, fibers, organic acids, essential oils, flavonoids and carotenoids [3]. Among these compounds, flavonoids have received considerable attention because of their beneficial effects as antioxidants in the prevention of human diseases [4]. In addition, flavonoids have been found to exhibit a wide spectrum of pharmacological properties, including antiallergic, anti-inflammatory, antidiabetic, antiviral and antineoplastic activities [5,6].

Recent R&D efforts aim at converting the potential of wastes into profitable products creating new segments of production and offsetting the disposal costs.

The growing interest for the utilization of phytochemicals as raw materials in cosmetic, pharmaceutical and nutraceutical preparations has urged researchers to investigate several methodologies for the extraction and purification of phenolic compounds from citrus peels. They include solvent extraction [7], ultrasound-assisted extraction [8], enzyme-aided extraction [9], supercritical fluid extraction [10], resin-based extraction [11] and alkaline extraction [12]. These extraction methods are characterized by some drawbacks, including the degradation of the target compounds due to high temperatures and long extraction times (as in solvent extractions) and health-related risks. Compared to conventional methods, membrane-based technologies offer several advantages in terms of high separation efficiency, low energy requirements, mild operating conditions, no additives, the absence of a phase transition, simple equipment and easy scale-up [13].

The potentialities of membrane unit operations in different areas of the agro-food production are well known. New perspectives result from the combination of different membrane unit operations or between membrane operations and conventional separation technologies (*i.e.*, adsorption, centrifugation, evaporation). These combinations offer interesting benefits that cannot be achieved when developed as one concept. In addition, the development of hybrid processes, within the logic of the process intensification strategy, offers new and much more opportunities in terms of competitiveness, improvement of quality, process or product novelty and environmental friendliness [14].

The integration of pressure-driven membrane operations, such as ultrafiltration (UF) and nanofiltration (NF), has been demonstrated as an attractive alternative for producing, at low temperatures, concentrated anthocyanin extracts from *Aronia* fruits (black chokeberry) [15] and plants [16]. The fractionation of proanthocyanidins from winery extracts [17], the recovery of phenolic compounds from bergamot juice [18], olive mill wastewaters [19,20,21] and artichoke wastewaters [22] are others interesting applications of integrated membrane operations in this field.

In this work, the feasibility of an integrated process based on the use of UF, NF and osmotic distillation (OD) processes for the recovery and concentration of flavonoids from press liquors was investigated. The performance of selected membranes was evaluated in terms of productivity and retention towards flavanones and anthocyanins, which are highly recognized for their antioxidant capacities.

## 2. Materials and methods

### 2.1. Orange Press Liquor

Citrus press liquors from blood orange peel processing were supplied by Citrech Snc (Rosarno, Reggio Calabria, Italy). Liquors were left overnight at room temperature to let the majority of the cloud particles settle out. A partially clear liquor was recovered after decanting the cloud layer and filtration with a nylon cloth. The liquor was then stored at −17 °C and defrosted before use.

### 2.2. UF Experimental Set-Up and Procedures

The raw press liquor was clarified by UF. Cross-flow UF experiments were performed in a pilot unit (Verind S.p.A., Milan, Italy) equipped with a polysulfone hollow fiber membrane module supplied by China Blue Star Membrane Technologies, Co., Ltd. (Beijing, China). Characteristics of the membrane module are reported in Table 1. The equipment consists of a stainless steel feed tank, a feed pressure pump, two manometers (0–40 bar) located at the inlet (*P_in_*) and outlet (*P_out_*) of the membrane module and a magnetic flow meter for measuring the axial feed flow rate (*Q_f_*). A tube and shell heat exchanger, placed after the feed pump, was used to maintain the temperature of the feed solution constant. A data acquisition system, permitting the continuous monitoring of the transmembrane pressure (TMP) and of the axial feed flow rate, was connected to the UF plant. The permeate flux was calculated according the following equation:

(1) $$J_{p}=\frac{V_{p}}{t\cdot A}$$

where *V_p_* is the permeate volume collected during the time interval *t* and *A* the membrane surface area.

**Table 1:** Characteristics of the UF membrane module.

Type	DCQ III-006
Configuration	Hollow fiber
Membrane material	Polysulfone
Module dimension (mm)	90 × 522
Operating pressure (bar)	1–1.5
Operating temperature (°C)	0–40
Operating pH	2–13
Inner fiber diameter (mm)	2.1
Membrane surface area (m^2^)	1.2
Nominal molecular weight cut-off (kDa)	100

The UF system was operated at a TMP of 0.54 bar, a *Q_f_* of 500 L·h^−1^ and a temperature of 25 ± 1 °C according to a batch concentration configuration, collecting separately the permeate stream and recycling back the retentate stream to the feed tank.

UF experiments were performed by using, on average, 40 L of press liquor until the feed volume was reduced to 7.6% of the original volume, corresponding to a volume reduction factor (VRF) of 13.4.

The VRF was calculated according to Equation (2):

(2) $$VRF=\frac{V_{f}}{\left(V_{f}-Vp\right)}$$

where *V_f_* is the total feed volume and *V_p_* the volume of collected permeate.

### 2.3. NF Experimental Set-Up and Procedures

The clarified fraction coming from the UF process was concentrated by NF. NF experiments were performed by using a laboratory pilot unit manufactured by Matrix Desalination Inc. (Fort Lauderdale, FL, USA). The equipment consists of a feed tank, a cooling coil working with tap water, a high pressure pump, a stainless steel housing, a permeate flowmeter and a pressure control system. The plant was equipped with a spiral wound membrane module (Nadir NF-PES 10, 2440 C) supplied by Microdyn-Nadir (Wiesbaden, Germany) (Table 2).

**Table 2:** Characteristics of the NF membrane module.

Type	Nadir NF-PES 10
Configuration	Spiral wound
Membrane material	Polyethersulfone
Maximum operating pressure (bar)	40
Maximum operating temperature (°C)	50
Operating pH	2–9
Membrane surface area (m^2^)	1.6
Na_2_SO_4_ rejection (%)	25–50
NaCl rejection (%)	5–15

NF experiments were performed according to a batch concentration configuration in selected operating conditions (TMP, 8 bar; *Q_f_*, 400 L·h^−1^; temperature, 20 ± 1 °C) up to a VRF of 5.

### 2.4. OD Experimental Set-Up and Procedures

The NF retentate was concentrated by OD by using a laboratory plant supplied by Hoechst-Celanese Corporation (Wiesbaden, Germany). The plant consists of: two magnetic drive gear pumps for the circulation of both preconcentrated liquor and stripping solution in the shell side and in the lumen side (tube side) of the OD membrane module, respectively; four pressure gauges in order to register inlet and outlet pressures for both tube side and shell side streams; a digital balance (Gibertini Elettronica, Milan, Italy), placed under the juice tank; two flow-meters for the measure of brine solution and feed flow rate, respectively.

The plant was equipped with a Liqui-Cel^®^ Extra-Flow 2.5 × 8 inches membrane contactor supplied by Hoechst-Celanese Corporation (Wiesbaden, Germany). The OD membrane module is constituted by hydrophobic hollow fiber membranes with external and internal diameters of 300 µm and 220 µm, respectively. Other characteristics of the membrane module are reported in Table 3.

**Table 3:** Data sheet of the Liqui-Cel^®^ Extra-Flow 2.5 × 8ʺ membrane contactor.

Fibers Characteristics	Fiber Type Celgard^®^ Microporous Polypropylene Hollow Fiber
*Cartridge Operating Limits*	
Maximum Transmembrane Differential Pressure	4.11 kg/cm^2^
Maximum Operating Temperature Range	40 °C
*Cartridge Characteristics*	
Cartridge Dimensions (D × L)	8 cm × 28 cm
Effective Surface Area	1.4 m^2^
Effective Area/Volume	29.3 cm^2^
Fiber Potting Material	Polyethylene

The NF retentate with an initial concentration of 32 g total soluble solids (TSS) 100 g^−1^, was pumped through the shell side of the membrane module, while in the tube side flowed a 10.2 mol·L^−1^ calcium chloride dihydrate (Fluka Chemie GmbH, Buchs, Switzerland) solution, in a counter current mode. Both solutions were re-circulated back to their reservoirs, after passing through the contactor, at a temperature of 28 ± 2 °C. Flow rates of feed and stripping solutions were fixed at 66 L·h^−1^ and 26 L·h^−1^, respectively.

The OD system was operated with a slightly higher pressure on the shell side than the lumen side in order to avoid the leakage of the brine strip into the product. The operating TMP was fixed at 0.28 bar.

The flow rate of the extracted water, at various points during the concentration process, was calculated by measuring the weight loss of the feed over the time by a digital balance. Evaporation fluxes (*J_w_*) were calculated from flow rate values divided by the membrane surface area (1.4 m^2^).

### 2.5. Analytical Measurements

Feed, permeate and retentate samples collected in the investigated membrane process were analyzed for total soluble solids (TSS), total flavanones, total anthocyanins content and anthocyanin compounds.

The rejection (*R*) of the UF and NF membranes towards the investigated compounds was determined as:

(3) $$R=(1-\frac{C_{p}}{C_{f}})\times 100$$

where *C_p_* and *C_f_* are the concentration of a specific component in the permeate and feed, respectively.

#### 2.5.1. TSS and Suspended Solids Measurement

The TSS content was determined by an Abbe refractometer Bellingham+Stanley 60/DR (Bellingham+Stanley Ltd., Kent, UK). Prior to each set of measurements, the instrument was calibrated at 0 g TSS 100 g^−1^ by using deionized water. Measurements were made in triplicate at 20 °C. Standard deviations (SD) were within 0.5 g TSS 100 g^−1^.

The suspended solids content was determined in relation to the total liquor weight (% w/w) by centrifuging 45 mL of a pre-weighed sample, at 2000 rpm for 20 min, and the weight of settled solids was determined after removing the supernatant.

#### 2.5.2. Determination of Flavanones

The content of flavanones was measured by using a modified colorimetric method [23]. The diluted sample (0.5 mL) was added to a test tube containing 3.5 mL of absolute ethanol. After the addition of 4 mL of 90% diethylene glycol and mixing, the reaction was initiated by adding 0.1 mL of 4 M NaOH. Absorbance at 420 nm was measured after 10 min of incubation at 40 °C, by using a UV-Vis recording spectrophotometer (UV-160A, Shimadzu Scientific Instruments, Inc., Kyoto, Japan). Hesperidin was used as the standard, and the total flavanone content was expressed as g hesperidin equivalents (gHE L^−1^). Measurements were made in triplicate. Standard deviations (SD) were within ±2%.

#### 2.5.3. Determination of Total Anthocyanins

The determination of the total anthocyanins was carried out by a colorimetric method [24,25]. Spectrophotometric analyses were performed under the following conditions: to 5 mL of liquor were added 40 mL of an EtOH/HCl mixture previously prepared by mixing 79.3 mL of anhydrous ethyl alcohol with 20.3 mL of HCl (37%). The absorbance was measured at 535 nm by using the UV-160A Shimadzu instrument. The calibration curve was obtained by measuring the absorbance of standard solutions of pure cyanidin-3 glucoside. The anthocyanin concentrations were calculated using the extinction coefficient 1018.3. Measurements were made in triplicate. Standard deviations (SD) were within ±2%.

#### 2.5.4. HPLC Determination of Individual Anthocyanic Compounds

The concentration of individual anthocyanin compounds was determined by HPLC analyses by using an HPLC-system (Agilent 1100 series, Agilent Technologies, Waldbronn, Germany), equipped with a Luna C18 column (250 mm × 4.6 mm, 5 µm, Phenomenex, Torrance) and an UV detector. The following conditions were used: V = 1 mL·min^−1^; temperature = 25 °C; λ = 518 nm. The mobile phase was a mixture of H_2_O/HCOOH (9:1) as Solvent A and H_2_O/HCOOH/CH_3_CN (4/1/5) as Solvent B. Anthocyanins separation was achieved by using the following linear gradient: starting condition, 88% A, 12% B; 26 min, 70% A, 30% B; 35 min, 100% B; 43 min, 88% A, 12% B; 46 min 88% A, 12% B.

Clarified samples coming from the UF process were diluted 1:10, while NF and OD retentates were diluted 1:100. Anthocyanins were identified by matching the retention time and their spectral characteristics against those of standards (cyanin chloride, myrtillin chloride, cyanidin 3-glucoside chloride, peonidin-3-glucoside chloride). For the quantitative analysis, a standard calibration curve was obtained by plotting the peak area against different concentrations of each standard compound. The deviation of each measurement was 2% from the average value.

## 3. Results and Discussion

### 3.1. Ultrafiltration

**Figure 1:**
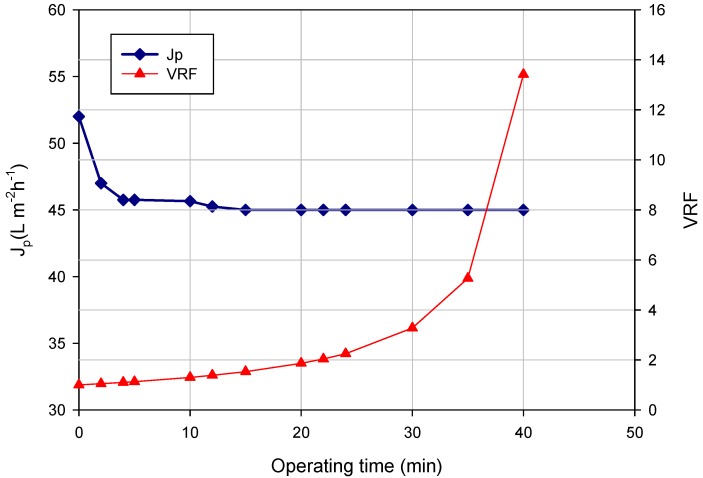
UF of orange press liquor. Time course of permeate flux (*J_p_*) and VRF (operating conditions: transmembrane pressure (TMP), 0.54 bar; *Q_f_*, 500 L·h^−1^; temperature, 25 °C).

Figure 1 shows the time course of the permeate flux and VRF for the clarification of the orange press liquor in the selected operating conditions. Experimental data refer to the treatment of 39 L of raw press liquor and the production of about 36 L of clarified liquor (final VRF, 13.4).

The flux decline can be attributed to the accumulation of liquor components in the pores and on the membrane surface, which are responsible for membrane internal pore fouling and gel formation phenomena, respectively.

The initial permeate flux of about 52 L·m^−2^·h^−1^·bar^−1^ reached a steady-state value of about 45 L·m^−2^·h^−1^·bar^−1^ when the VRF was 1.4. Lower steady-state fluxes (2.7 kg·m^−2^·h^−1^) were observed when the same UF membrane was used to clarify the depectinized blood orange juice at higher TMP values (0.8 bar) [26].

### 3.2. Nanofiltration

**Figure 2:**
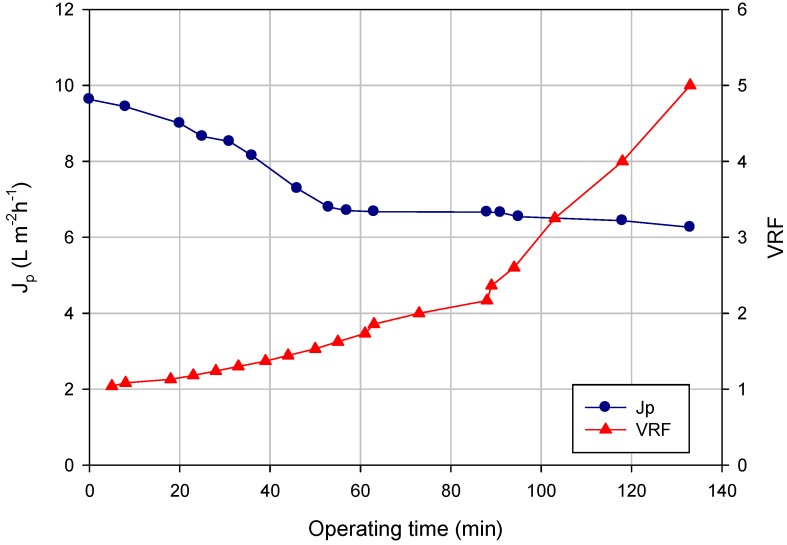
The NF of orange press liquor. The time course of permeate flux and VRF (operating conditions: TMP, 8 bar; *Q_f_*, 400 L·h^−1^; temperature, 20 °C).

The clarified liquor was pre-concentrated by NF in selected operating conditions (TMP, 8 bar; temperature, 20 °C). Figure 2 shows the time evolution of the permeate flux and of the VRF referring to an NF test carried out according to the batch concentration mode in which, starting from 26 L of clarified liquor, 20.8 L of permeate were produced (final VRF, five). The initial permeate flux of 9.6 L·m^−2^·h^−1^ decreased gradually in the first 60 min; then the permeate flux reached a steady-state permeate value of about 6 L·m^−2^·h^−1^ until to the final VRF. The flux decay can be attributed to the adsorption of specific compounds on the membrane surface or within membrane pores. In particular, the adsorptive fouling of phenolic compounds on PES membranes is influenced by the pore size, polar interactions (van der Waals, electron donor-acceptor interaction) and multiple hydrogen bonds towards the additive polyvinylpyrrolidone (PVP) used in the manufacture of PES membranes [27]. An increase of both the adsorbed amount and affinity for polyphenol binding to PES membranes with increasing PVP content was also reported by [28]. In addition, mixtures of polyphenols with other components, such as polysaccharides, could form aggregates having a strong contribution to adsorptive fouling of PES membranes [29].

In a previous work performed on the unclarified press liquor [30], the NF-PES 10 membrane exhibited an initial permeate flux of 10.4 L·m^−2^·h^−1^ and a steady-state permeate flux of 3.4 L·m^−2^·h^−1^ when the system was operated at a TMP of 6 bar and a temperature of 20 °C up to a final VRF of three. According to our data, the UF step allowed the application of high flow-rates and the maximization of yields during the NF treatment. Similar results were also obtained in the clarification and concentration of grape juice by an integrated UF-OD process [31].

### 3.3. Osmotic Distillationmol

**Figure 3:**
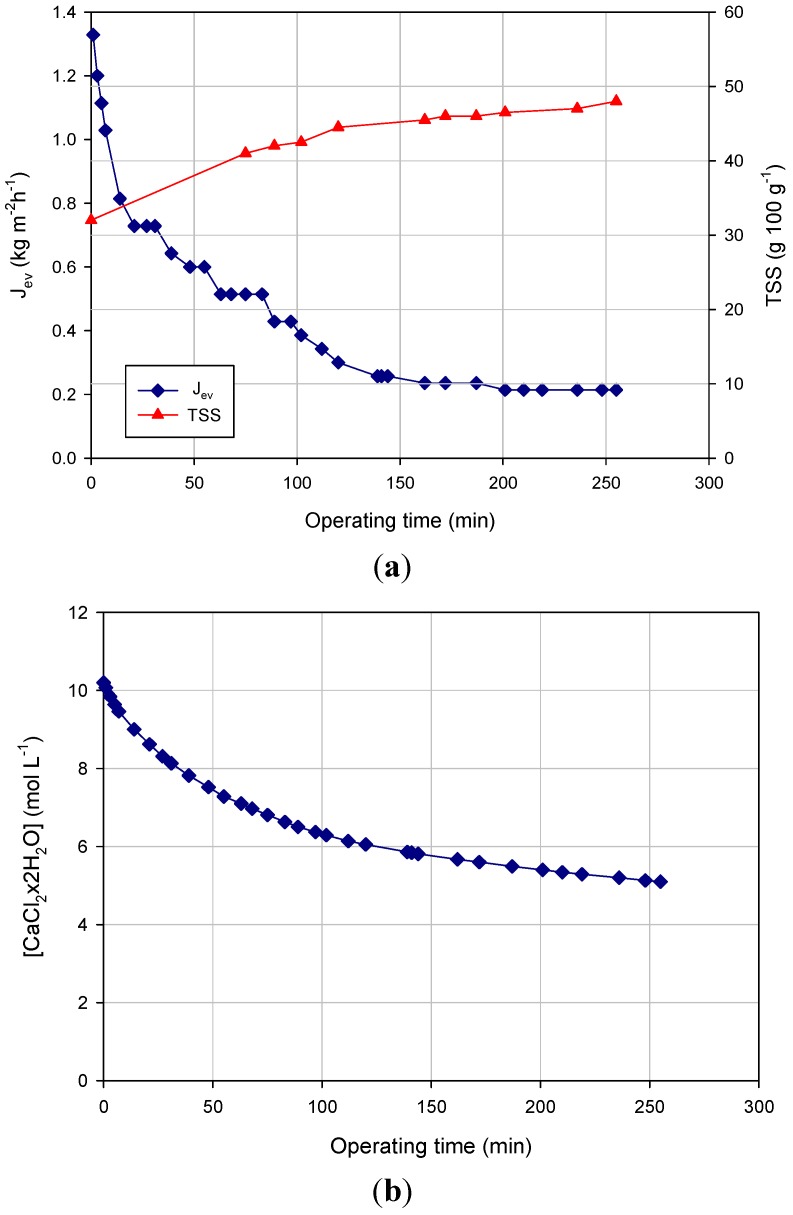
Concentration of NF retentate by OD. Time course of (**a**) evaporation flux and TSS; (**b**) stripping solution concentration (operating conditions: TMP, 0.28 bar; temperature, 28 ± 2 °C; *Q_f_*, 66 L·h^−1^; *Q_b_*, 26 L·h^−1^).

The NF retentate was finally concentrated by OD. Figure 3 shows the experimental results related to the concentration of the NF retentate within a closed loop from 32 g TSS 100 g^−1^ up to 47 g TSS 100 g^−1^. At first, the brine concentration was 10 mol·L^−1^, producing an evaporation flux of 1.32 kg·m^−2^·h^−1^. The evaporation flux decreased gradually during the process to reach a final value of 0.21 kg·m^−2^·h^−1^ corresponding to a final concentration of 47 g TSS 100 g^−1^ (Figure 3a). The decrease of evaporation flux can be mainly attributed to the dilution of the brine solution. In particular, a 43% decrease of the brine concentration in the range 0–150 min (from 10.2 mol·L^−1^ to 5.8 mol·L^−1^) (Figure 3b) produced a water vapor flux decay of about 83%. This result shows the strong influence of the brine concentration on the evaporation flux and, consequently, on the driving force of the OD process.

In the range 150–250 min, the evaporation flux remained unchanged despite the increase in the TSS concentration of the liquor. Adversely, studies performed on the concentration of fruit juices and sucrose solutions by OD showed a decreasing of the evaporation flux when the TSS concentration was higher than 30–35 g·100 g^−1^ [32,33].

### 3.4. Retention of Flavonoids

The UF membrane removed all suspended solids from the raw press liquor, producing a clear solution with a content of flavanones and anthocyanins similar to that of the initial liquor. Indeed, the rejection of the UF membrane towards flavanones and anthocyanins was lower than 1%. The clarified liquor contained almost all of the soluble solids of the initial feed (10 g·100 g^−1^ if compared to 10.1 g·100 g^−1^ of the raw press liquor) (Table 4). The TSS content appeared to be higher in the retentate stream than in the clarified liquor: this phenomenon can be attributed to the high suspended solid content of the feed solution, which can interfere with the measurements of the refractive index.

The NF process produced a pre-concentrated liquor with 32 g TSS 100 g^−1^ from the clarified liquor. During the NF process, phenolic compounds were concentrated in the retentate side. The rejection of the NF membrane towards flavanones and anthocyanins was 97.4% and 98.9%, respectively. Similar rejections for the anthocyanins were measured by Cissé *et al.* [16] in the treatment of *Roselle* extract with the same NF membrane, but in flat-sheet configuration (designed as NP010).

**Table 4:** Analytical evaluation of anthocyanins and flavanones in samples of press liquor coming from the UF treatment.

Sample	Suspended Solids (%)	TSS (g·100 g^−1^)	Total Flavanones (g·HE·L^−1^)	Total Anthocyanins (g·L^−1^)
Feed UF	7.13	10.1	22.850	3.160
Permeate UF	0	10.0	22.801	3.065
Retentate UF	93.6	10.2	22.900	3.380

The high rejection for anthocyanin compounds can be explained assuming that anthocyanins, unlike other subgroups of flavonoids with a similar C6-C3-C6 skeleton, have a positive charge in their structure at acidic pH. The pH of the clarified press liquor is about three; at this pH value, the selected membrane exhibits a positive charge. In particular, data reported in the literature indicate a zeta potential of 1 mV at pH 3 for the NFPES10 membrane [34]. Consequently, the electrostatic repulsion contributes to the high rejection of the NF membrane towards anthocyanins.

**Figure 4:**
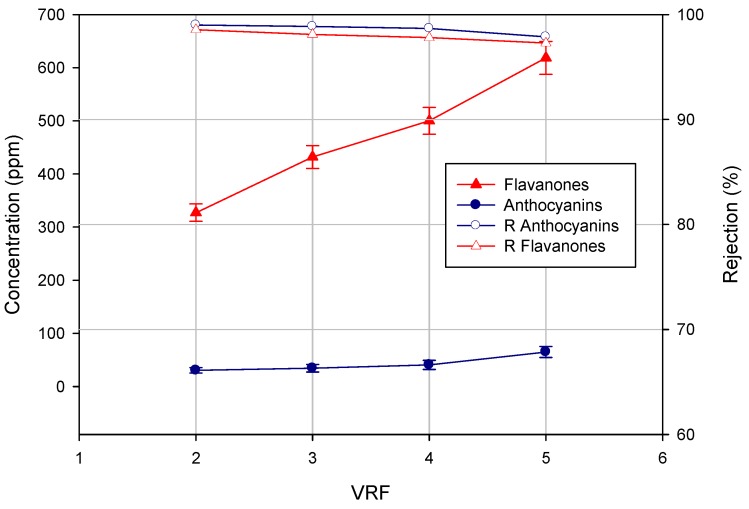
Concentration of flavanones and anthocyanins in the NF permeate as a function of VRF.

As shown in Figure 4, the concentration of both flavanones and anthocyanins in the permeate stream increased by increasing the VRF. Consequently, the rejection values decreased slightly with increases in the VRF. These results are representative of filtration processes in the batch concentration configuration: the increase of VRF leads to an increase of the concentration of phenolic compounds in the feed solution, which facilitates their transport to the NF membrane, thus increasing slightly their concentration in the permeate stream according to the diffusion control theory [35]. Similar results were obtained in the removal of pharmaceutically active compounds from drinking water sources [36,37].

**Figure 5:**
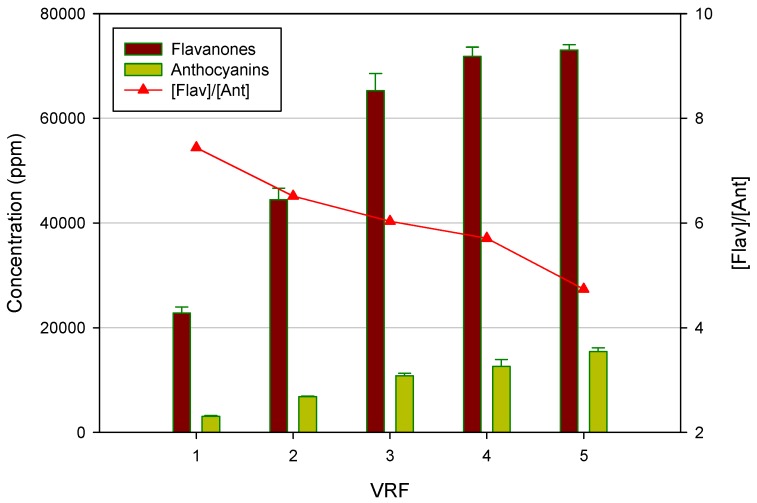
Concentration of flavanones and anthocyanins in the NF retentate as a function of VRF.

Figure 5 shows the effect of VRF on the concentration of both flavanones and anthocyanins in the NF retentate. As expected, the concentration of both compounds increased by increasing the VRF. Interestingly, the ratio between flavanones and anthocyanins decreased when the VRF was raised. In particular, the initial flavanones/anthocyanins ratio of 7.43 was reduced up to 4.73 at VRF five. This result is of particular interest for the exploitation of the product at an industrial level. Indeed, the clarified press liquor is configurable as an intermediate product characterized by a low coloring power and a strong bittering capacity, due to the higher concentration of flavanones. In order to use the product as a food coloring, it is necessary to balance the flavonoid content in relation to anthocyanin compounds, so as to interfere as little as possible with the organoleptic characteristics of the product under staining. Therefore, the control and the optimization of the VRF in the NF process can be exploited to modify the ratio between flavanones and anthocyanins in order to characterize the final product for its bittering capacity and coloring power.

In Table 5, the total content of anthocyanins, flavanones and soluble solids in the retentate fractions of both of the NF and OD processes is reported. The concentration factor of anthocyanins in the final OD retentate was in agreement with that of the TSS content due to the water removal. The lowest concentration factor reached for flavanones in the NF retentate could be attributed to the adsorption phenomena of these compounds on the NF membrane.

**Table 5:** Analytical evaluations of total anthocyanins, flavanones and TSS in samples of clarified liquor from NF-OD treatments.

Sample	TSS	Total Anthocyanins	Total Flavanones
	(g·100 g^−1^)	(g·L^−1^)	(g·HE·L^−1^)
Feed NF	10.0	3.065	22.801
Retentate NF	32.0	15.425	72.160
Retentate OD	47.0	20.978	98.250

In Table 6, HPLC determinations of different anthocyanin compounds in the clarified press liquor and NF and OD retentates are reported. As can be seen, cyanidin-3-glucoside chloride was the predominant anthocyanin compound of the clarified press liquor (feed NF), followed by myrtillin chloride and peonidin-3-glucoside chloride.

**Table 6:** Analytical evaluations of individual anthocyanins in samples of clarified liquor from NF-OD treatments.

Sample	Cyanin Chloride	Cyanidin-3-glucoside Chloride	Myrtillin Chloride	Peonidin-3-glucoside Chloride
	(ppm)	(ppm)	(ppm)	(ppm)
Feed NF	84.83	255.6	55.23	53.04
Retentate NF	–	1304.34	300.51	213.20
Retentate OD	639.19	1787.70	400.63	399.67

**Figure 6:**
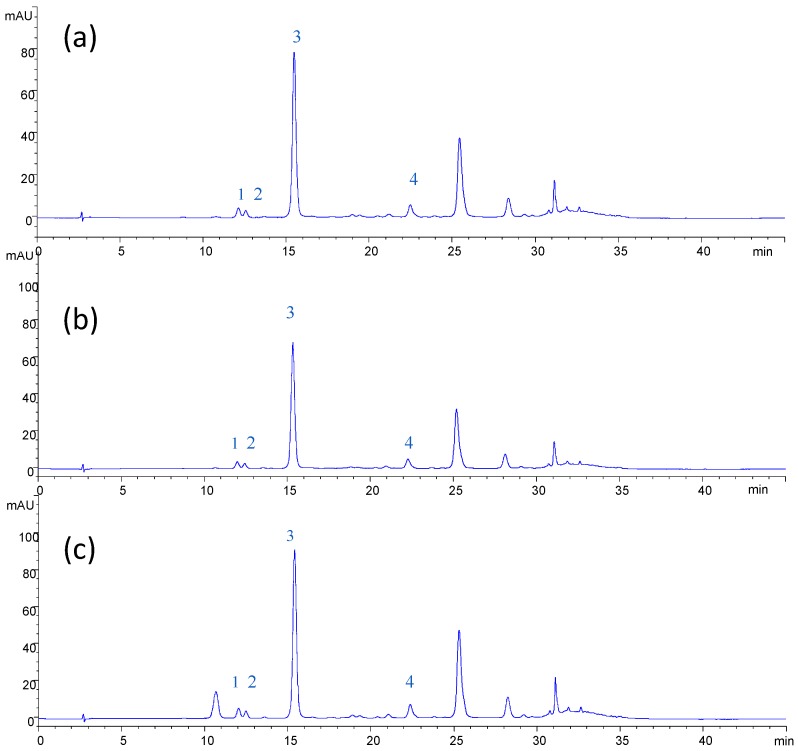
HPLC chromatogram of anthocyanins. (**a**) Clarified press liquor; (**b**) NF retentate; (**c**) OD retentate. Peaks: 1, cyanin chloride; 2, myrtillin chloride; 3, cyanidin-3-glucoside chloride; 4, peonidin-3-glucoside chloride.

The chromatographic profiles of anthocyanins in the samples of NF feed, NF and OD retentates (Figure 6) indicated that anthocyanin compounds are well preserved during the clarification and concentration of the press liquor with the selected membranes. These results confirm the possibility of producing at low temperatures preconcentrated extracts without thermal damage before final concentration by OD [16]. On the other hand, a strong degradation of anthocyanin pigments in the pomegranate juice was observed when the juice is concentrated by thermal evaporation accomplished by the presence of significant levels of 5-hydroxymethyl furfural (an indicator of the potential browning of the juice) and by the reduction of minerals [38].

**Figure 7:**
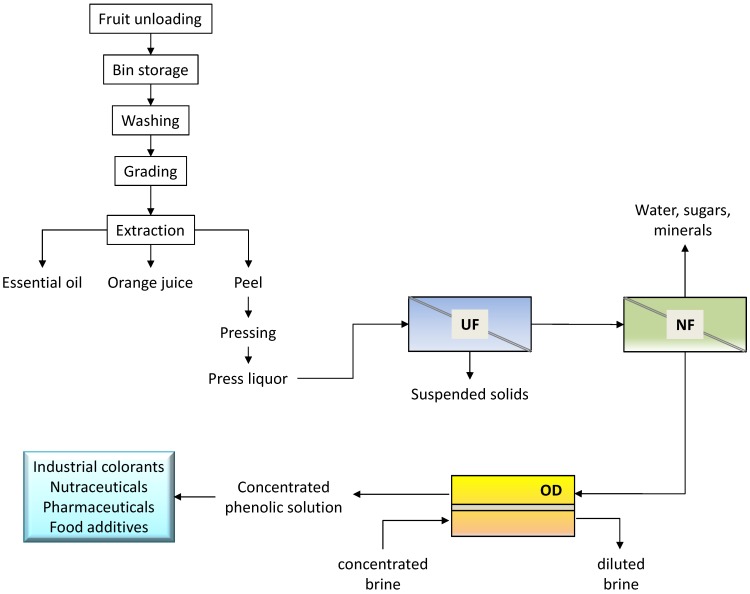
Schematic of the integrated membrane process proposed for the recovery of flavonoids from orange press liquor.

An integrated membrane process for the recovery and concentration of flavonoids from orange press liquor was proposed on the basis of the experimental results (Figure 7).

The preliminary UF step allows one to remove suspended solids from the raw solution, producing a clear permeate in which most part of the flavonoids was recovered. Flavanones and anthocyanins are pre-concentrated in the NF step with a production of a permeate stream (with a TSS content of 4.5 g·100 g^−1^) containing sugars and minerals. The final treatment of the NF retentate by OD produces a concentrated solution of great interest for food and pharmaceutical applications. Indeed, flavanones are highly recognized for their pharmacological properties (favorable effect on capillary fragility and treatment of inflammatory states) arising from their antioxidant activity. In addition, the extract can be used in food coloring, so avoiding the use of artificial colorants.

## 4. Conclusions

An integrated membrane process based on the use of UF, NF and OD operations was proposed for recovering flavonoids from the liquid wastes of orange juice production.

Suspended solids were completely removed from the raw press liquor by UF, producing a clear liquor in which most part of the flavonoids were recovered. These compounds were highly retained by the NF membrane, and their concentration in the NF retentate increased by increasing the VRF of the process.

The ratio between flavanones and anthocyanins in the NF retentate was affected by the VRF of the process: consequently, the bittering capacity and coloring power of the final product can be optimized through the selection of specific VRF values.

The NF membrane allowed for the removal also of part of the sugars (the rejection of the NF membrane towards TSS was of the order of 55%) and minerals.

The final step of OD allowed for obtaining a concentrated flavonoid-based solution operating in conditions of low mechanical and thermal damage.

The proposed process allows one to redesign the traditional flow-sheet of the citrus processing industry with significant advantages in terms of the reduction of environmental impact and depollution costs, recovery and reuse of high added-value compounds and the reduction of water consumption. In addition, preconcentrated and concentrated extracts were produced at low temperatures without thermal damage to the compounds of interest, offering interesting perspectives for the use of these products as natural colorants and/or for nutraceutical applications.
